# Incidental amniocele in a case of antepartum haemorrhage

**DOI:** 10.4102/sajr.v24i1.1817

**Published:** 2020-02-06

**Authors:** Sheree C. Gray, Jacobus A. Pienaar, Zelia Sofianos, Jacob Varghese, Ilonka Warnich

**Affiliations:** 1 Department of Diagnostic Radiology, Tshepong Hospital Complex, Klerksdorp, South Africa; 2 Department of Diagnostic Radiology, School of Clinical Medicine, Faculty of Health Sciences, University of the Witwatersrand, Johannesburg, South Africa

**Keywords:** amniocele, contained uterine rupture, foetal imaging, antepartum haemorrhage, placenta previa

## Abstract

An amniocele, or contained uterine rupture, is a phenomenon in which there is herniation of the amniotic sac through a uterine defect, secondary to various causes. It is associated with severe morbidity and mortality. This case presents the findings in a 36-year-old female at 29 weeks gestation who was initially managed as antepartum haemorrhage secondary to placenta previa, based on ultrasound. Upon further imaging, an amniocele was diagnosed. This case report illustrates the importance of early identification of this life-threatening condition.

## Introduction

An amniocele, otherwise known as a contained uterine rupture, is a rare phenomenon in which there is herniation of the amniotic sac through a uterine defect. This rare entity can be associated with severe morbidity and mortality for both mother and foetus. The causes for an amniocele include certain invasive obstetric/gynaecological procedures or alternatively it may be an idiopathic occurrence. It is more commonly identified in developing countries where these procedures are frequently performed by inexperienced or inadequately trained healthcare professionals.

## Case presentation

A 36-year-old pregnant female patient presented to the antenatal clinic with a history of acute lower abdominal pain and associated vaginal bleeding, reporting passing drops of blood without passage of clots. On clinical examination, the patient was haemodynamically stable with no significant abnormalities identified. Background clinical history revealed that she was a booked patient and compliant with antenatal follow-up visits. This was her fourth pregnancy, with two previous normal vaginal deliveries at term. Records also revealed a previous termination of pregnancy, carried out 5 years prior at 16 weeks of gestational age. The termination was conducted as an inpatient procedure, likely involving invasive uterine instrumentation; however, further details were not available.

Biochemistry revealed a normal full blood count. She was known to be Human Immunodeficieny Virus (HIV) positive, with a CD4 count of 98 and a viral load of 212, as well as Rapid Plasma reagin (RPR) negative, Rhesus positive and Hepatitis B surface antigen negative. She had no other known co-morbidities. During the current pregnancy, the patient had been repeatedly treated with antibiotics for recurrent urinary tract infections with culture-proven *Escherichia coli.* She also had a prolonged hospital admission for more than 2 weeks after presenting with vaginal bleeding, and was assessed with a threatened miscarriage. At the time, the bleeding resolved spontaneously.

An initial abdominal ultrasound at 21 weeks of gestational age had identified a low-lying placenta. A subsequent follow-up obstetric ultrasound documented an intrauterine singleton at approximately 29 weeks of gestational age, with a vertex presentation. Placenta previa was noted, with the suspicion of morbid adherence. No evidence of abruptio placentae was found. The patient was subsequently admitted to hospital for monitoring and further workup, with a diagnosis of antepartum haemorrhage as a complication of placenta previa.

**FIGURE 1:**
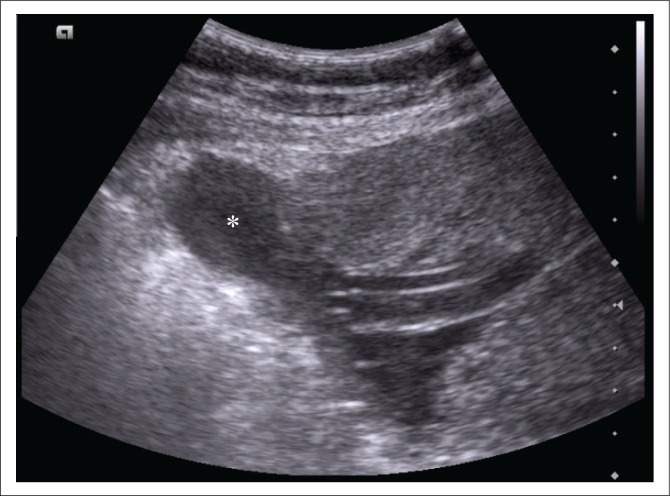
Transverse abdominal ultrasound demonstrating a well-circumscribed, anechoic structure (asterisk), adjacent to placental tissue, occupying the uterine fundus on the right. Communication with the uterine cavity is evident, in keeping with the amniocele demonstrated on magnetic resonance imaging.

**FIGURE 2:**
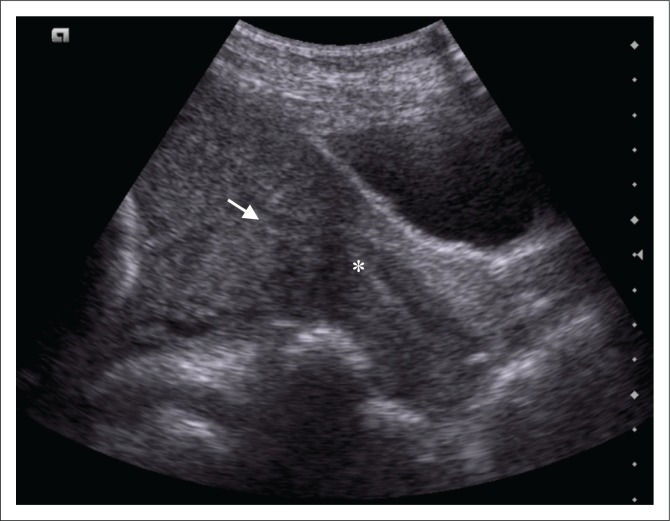
Longitudinal abdominal ultrasound demonstrating placental tissue (white arrow) overlying the internal ostium (os) of the cervix (asterisk).

Prior to magnetic resonance imaging (MRI), a repeat transabdominal ultrasound was performed by the radiology department. This demonstrated a right-sided parauterine cystic lesion, not previously noted, with apparent communication with the uterine cavity (Figure 1). Placental tissue was confirmed overlying the internal cervical ostium (os) but appeared separate from the main bulk of the placenta (Figure 2).

**FIGURE 3:**
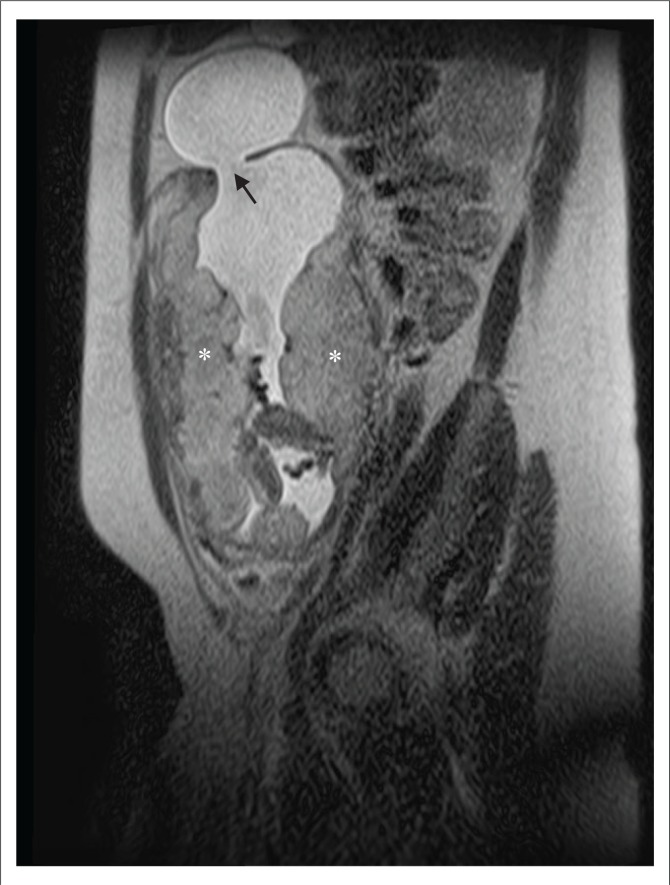
Sagittal T2-weighted magnetic resonance imaging demonstrating a defect in the myometrium of the uterine fundus through which part of the amniotic sac has herniated (arrow). Placental tissue could be seen lining the anterior and posterior uterine walls, and extending to the fundus anteriorly (asterisks).

**FIGURE 4:**
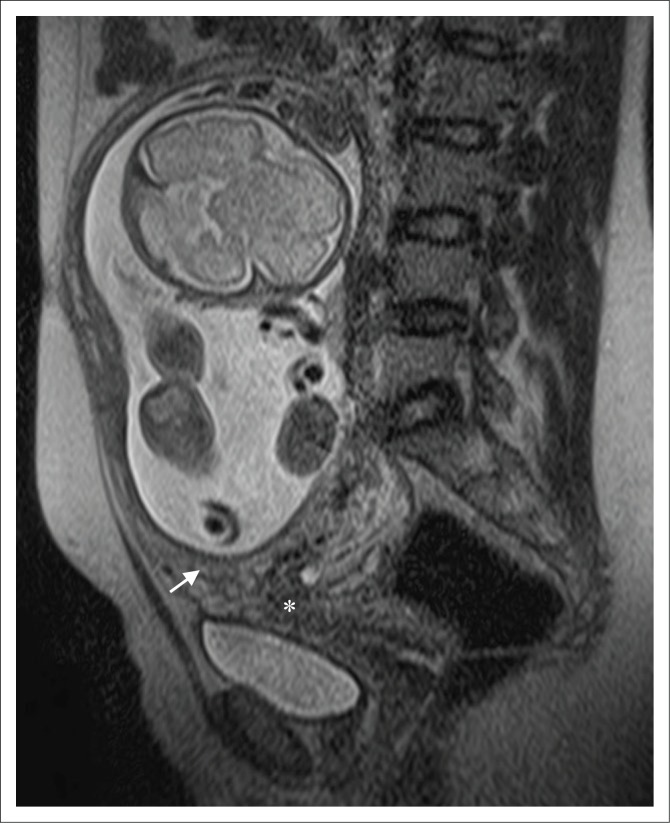
Sagittal T2-weighted magnetic resonance imaging demonstrating low-lying placental tissue (white arrow), separate from the main bulk of the placenta, with complete coverage of the internal ostium (os) of the cervix (asterisks).

Magnetic resonance imaging was subsequently performed to assess for abnormal placental invasion. It confirmed the presence of an intrauterine singleton pregnancy and showed the major bulk of the main placenta situated along the right uterine wall, extending anteriorly and posteriorly. The placental thickness and signal intensity was appropriate for the gestation, with a normal central cord insertion. A secondary, separate placental component was also identified more inferiorly in the lower segment, extending from the posterior wall to cover the internal cervical os. These features are in keeping with a succenturiate placental lobe, which, in this case, caused major placenta previa. No features of morbidly adherent placenta (MAP) were identified. However, MRI confirmed the incidental ultrasound finding of a defect in the right superior fundal myometrium, through which a large sac containing amniotic fluid had herniated (Figures 3 and 4). There was no herniation of foetal parts or cord elements. Additionally, there was focal thinning of the remaining fundal myometrium with loss of the normal trilaminar appearance. It was contained by a thin, regular T2-hypointense rim representing an intact overlying serosal layer, showing no communication with the abdominal cavity. The dimensions of the sac measured 67 mm × 48 mm × 66 mm. These findings are in keeping with amniocele formation, or contained uterine rupture. Intrauterine amniotic fluid volume remained normal with no oligohydramnios.

The subsequent plan of obstetric care was to manage the patient conservatively and to deliver the baby via elective caesarean section at 34 to 36 weeks of gestational age. Intra-operatively, myometrial dehiscence with a focal protrusion of the amniotic sac was noted. This defect was closed surgically with no complications. No neonatal complications were documented. During the post-partum period, the patient developed a wound haematoma but was subsequently lost to follow-up.

### Ethical consideration

This article followed all ethical standards for a research.

## Discussion

An amniocele is defined as the ‘herniation of the amniotic sac through a uterine defect’.^1^ It is, more simply, a contained uterine rupture with the protrusion of amniotic contents – variably including parts of the foetus – through the uterine wall and into the peritoneal cavity. The presence of a uterine defect or weakness within the uterine wall is most often secondary to uterine scarring. This may result from previous uterine surgery, such as myomectomy or caesarean section, or as a complication of previous instrumentation of the uterus, such as dilatation and curettage. Rarely, it may be an idiopathic finding in an unscarred uterus.^2,3^

Amniocele formation is more commonly identified in developing countries where obstetric and gynaecological procedures are performed by healthcare workers who may not be experienced or adequately trained.^3^ There is a paucity of data documenting this rare entity, which is concerning, given that the consequences hereof could result in disastrous morbidity – and even mortality – for both patient and foetus.^4^ For this reason, it is imperative that this abnormality be detected as early as possible so as to guide the monitoring of both patient and foetus, as well as informing management decisions with the intention of minimising the risks of intrapartum complications.

There are both invasive and non-invasive approaches in the management of the amniocele, which could include the following: termination of the pregnancy, surgical uterine repair or, alternatively, vigilant radiological monitoring up until the time of delivery.^4^ Radiological monitoring could be performed using ultrasound or MRI at regular intervals in order to monitor for an increase in the size of the amniocele, detect further uterine wall dehiscence or observe for herniation of foetal parts.

Magnetic resonance imaging is not performed routinely in obstetric imaging, since it is time-consuming, expensive and not readily available. This is especially true for primary healthcare settings. An obstetric ultrasound performed by a competent user is sufficient in most scenarios and even superior due in part to improved spatial resolution.^5^ Magnetic resonance imaging, however, demonstrates superior soft tissue contrast resolution, which makes it an excellent adjunct investigation when further imaging of the placenta is required,^5^ a common referral indication as illustrated in this case report. Variations in placental location, thickness and morphology can be assessed, as well as increasing the diagnostic sensitivity and specificity of a MAP.

An accessory placental lobe is a variation in placental morphology, termed a succenturiate placenta. The term ‘bilobed placenta’ could be used alternatively when the placental components are equal in size. Bridging blood vessels can usually be demonstrated within the membranes connecting the accessory lobe. When the accessory placental tissue and associated hypervascular interconnecting membranes are located within the lower uterine segment, a careful evaluation for placenta previa or vasa previa should be performed. Rupture of these vessels could result in the grave consequence of foetal exsanguination.^5^

A low-lying placenta is defined as a placental location within the lower uterine segment, measuring a distance less than 2 cm from the internal os of the cervix. As soon as the cervical os is covered by the placenta, the term placenta previa is used. Although still in common use, the descriptors partial, marginal and complete placenta previa should be avoided. Apart from an increased risk of antepartum and intrapartum haemorrhage, placenta previa is considered a significant risk factor for the presence of a MAP. This potentially life-threatening placental variation represents a group of disorders with varying degrees of myometrial invasion – namely placenta accreta, increta and percreta; these describe direct myometrial contact, myometrial invasion and extension beyond the myometrium respectively.^5^

In addition, obstetric MRI could be used for evaluating foetal anatomy, as well as assist in the work up of other ambiguous ultrasound findings such as demonstrated in our reported case of an incidentally identified amniocele.

## Conclusion

Amniocele formation is a rare entity but may be encountered in the healthcare settings of a developing country such as South Africa, and imaging professionals should be aware of its existence. This case illustrates that the identification of one serious and potentially life-threatening pregnancy-related complication does not exclude the presence of other equally serious coexisting conditions, and that thorough investigation and imaging should be performed in all high-risk pregnancies. It also highlights the importance of a high index of suspicion for complications where risk factors are present such as advanced maternal age and previous uterine instrumentation, even in the absence of definitive clinical features.

## References

[1] Jo YS, Kim MJ, Lee GSR, Kim SJ A large amniocele with protruded umbilical cord diagnosed by 3D ultra-sound. Int J Med Sci. 2012;9(5):387–390. 10.7150/ijms.3383 22811613 PMC3399219

[2] Vimercati A, Del Vecchio V, Chincoli A, Malvasi A, Cicinelli E Uterine rupture after laparoscopic myomectomy in two cases: Real complication or malpractice? Case Rep Obstet Gynecol. 2017;2017:1–5. 10.1155/2017/1404815 PMC5750492 29423325

[3] Aiyekomogbon JO, Ojah SO, Shinkafi SM, Agom B A large amniocele through a fundal uterine defect diagnosed on 2D ultrasound imaging. Int J Case Rep Images. 2017;8(12):776–781. 10.5348/ijcri-2017121-CR-10860

[4] Report C, Azzaoui JEL, Chmichi N, et al A large amniocele with protruded right arm and hemithorax: A case report and review of the literature. World J Pharm Life Sci. 2019;5(3):28–36.

[5] Fadl S, Moshiri M, Fligner CL, Katz DS, Dighe M Placental imaging: Normal appearance with review of pathologic findings. RadioGraphics. 2017;37(3):979–998. 10.1148/rg.2017160155 28493802

